# Study protocol of a multi-center RCT testing a social-cognitive intervention to promote volunteering in older adults against an active control

**DOI:** 10.1186/s12877-019-1034-1

**Published:** 2019-01-24

**Authors:** Lisa M. Warner, Da Jiang, Alice Ming-Lin Chong, Tianyuan Li, Julia K. Wolff, Kee-Lee Chou

**Affiliations:** 1 MSB Medical School Berlin, Calandrelli Str. 1-9, 12147 Berlin, Germany; 2 The Education University of Hong Kong, 10 Lo Ping Rd, Tai Po, Hong Kong; 3 City University of Hong Kong, Tat Chee Ave, Kowloon Tong, Hong Kong; 4 Friedrich-Alexander-University (FAU) Erlangen-Nuremberg, Institute of Psychogerontology, Kobergerstr. 62, 90408 Nuremberg, Germany

**Keywords:** Volunteering, Older adults, Theory-based social-cognitive intervention, Randomized-controlled trial, Study protocol, Behavior change techniques

## Abstract

**Background:**

Volunteering could be a win-win opportunity for older adults: Links between volunteering and societal improvements as well as older adults’ own health and longevity are found in several observational studies. RCTs to increase volunteering in older adults are however sparse, leaving the question of causality unanswered. This study protocol describes a theory-based social-cognitive intervention with multiple behavior change techniques to increase volunteering among community-dwelling older adults in Hong Kong.

**Methods:**

In a parallel group, two-arm, randomized controlled trial, an initial *N* = 360 are assigned to receive either the volunteering intervention or the active control intervention (parallel content targeting physical activity). The primarily outcome measure is self-reported volunteering minutes per month at baseline, six weeks, three months and six months after the intervention. Participants in the treatment group are expected to increase their weekly volunteering minutes over time as compared to participants in the control group. Possible active ingredients of the intervention as well as mental and physical health outcomes of increased volunteering are investigated by means of mediation analyses.

**Discussion:**

Like many industrialized nations, Hong Kong faces a rapid demographic change. An effective psychological intervention to encourage retirees to engage in formal volunteering would alleviate some of the societal challenges a growing proportion of older adults entails.

**Trial registration:**

Primary Registry and Trial Identifying Number ChiCTR-IIC-17010349, secondary CCRB trial number CUHK_CCRB00543, registration date 2016/12/28.

**Electronic supplementary material:**

The online version of this article (10.1186/s12877-019-1034-1) contains supplementary material, which is available to authorized users.

## Background

Volunteering has often been described as a win-win-win activity for older adults, because the opportunity to “give back” not only enhances health and psychological well-being of volunteers but also brings benefits to the beneficiaries of volunteer activities as well as to the community as a whole [1]. These advantages of volunteering are of particular interest to societies that face demographic changes. Similar to other industrialized societies, Hong Kong observes longer life expectancies and declining fertility rates and therefore faces a previously unwitnessed demographic change. The number of adults aged 65+ is expected to more than double from 2016 with 1.16 million (16.6% of the general population) to 2.37 million (31.1% of the general population) in 2036 [2]. Compared to other industrialized societies, volunteering rates are, however, very low in Hong Kong with only 5.8%, compared to around 24% among older adults in the US and approx. 34% in Germany [3–6].

### Benefits of volunteering for older adults

Numerous cross-sectional and longitudinal studies find an association of volunteering with four expected pathways to health and longevity: Volunteering has consistently been found to be linked to mental health benefits, better social integration and social support, physiological resilience to stress as well as the performance of health behaviors [7–10]. In Hong Kong, a cross-sectional study found that post-retirement volunteering is positively associated with higher levels of self-efficacy, greater life satisfaction, and less psychological distress [11]. A voluntary home-visit program showed positive-effects of volunteering on the psychological well-being of volunteers in Hong Kong [12]. Both studies from Hong Kong and most studies incorporated into the reviews cited above are not sufficient to establish a causal link between volunteering and health as they lack randomization and comparison to a control group. Hence, older adults, who are better off, might as well be more likely to take up volunteering. To refute the argument of reversed causality, meta-analyses usually control for health status and various socio-demographic factors in order to account for the well-known effects higher socio-economic background and better health have on the likelihood to take up volunteering. Given the scarcity of randomized-controlled trials (RCTs) for volunteering, the question however remains, whether volunteering can actually be considered a health behavior itself or whether its links to health and longevity are methodological artefacts [8].

To date, the Experience Corps (EC) program is the first and only published RCT worldwide that examined the benefits of volunteering among older adults compared to a passive waiting-list control group [13–15]. The reported benefits of volunteering attained through the EC program are various and cover psycho-social (e.g. depressive symptoms, social network size; [13, 16]) cognitive (executive functioning, verbal learning, memory; [17, 18]) and physical health benefits (functional impairment, amount of walking, walking speed; [13, 16, 19, 20]) due to the uptake of volunteer activities in primary schools.

Even though the EC program was conducted in the field, it was highly structured and participants were randomized to specific volunteer tasks (e.g., supporting the school’s library, teaching conflict resolution to pupils). The approach of assigning older adults to specific and very narrow tasks contradicts the nature of volunteer activities. Different authors state “that older adults have highly diverse interests in causes and specific preferences for volunteer activities” ([21]; pp. 91–92) and “that participation in volunteer activities often relies on intrinsic motives such as altruism, personal development, and social responsibility, […]. Thus, it is not easy to match volunteers with tasks that could fulfil these factors.” ([6]; p. 318). Individuals’ freedom of choice to match their particular volunteer tasks with abilities, skills, expectations, interests, and time schedule is of utmost importance [22] and only given in naturalistic settings as compared to laboratory studies on helping behavior. Choosing, initiating and maintaining a suitable volunteer work, however, requires high motivation, organizational and social skills and therefore poses a challenge to older adults’ self-regulatory abilities. The challenge is therefore to enable older adults to select, initiate, and afterwards maintain volunteer work without being assigned to a specific task by a research team.

### The social-cognitive perspective on volunteering

Social-cognitive theories – known for their value in explaining (health) behavior – can contribute to the understanding and prediction of volunteer initiation and maintenance. According to the theory of planned behavior as adopted to volunteering, recent studies have shown that positive attitudes to volunteer work, self-efficacy for engaging in volunteerism, and social support for volunteering are positively associated with participation in volunteering work [23–26]. In other words, individuals who acknowledge the positive consequences of volunteering, report higher subjective abilities to carry out volunteering work, and receive the approval and support from their significant others are more likely to volunteer. Although these factors contribute to volunteering in cross-sectional and longitudinal surveys, experimental evidence for the utility of the theory of planned behavior to inform interventions for volunteering is scarce: There are few intervention studies based on social-cognitive theories for volunteering that have been conducted among adolescents [27] and parents of 4- to 17-year-old children [28] and only one study evaluated a volunteering intervention based on a social-cognitive theory among older adults. In this recent study in Germany, older adults were assigned at random to a social-cognitive intervention based on the health action process approach (HAPA; [29]) to promote volunteering, an active control intervention designed to motivate for physical activity, and a passive waiting list control group [30]. Participants were provided with information on the benefits of volunteering for older adults, reminded of their past successes, encouraged to set goals and to form implementation intentions, and exposed to positive volunteering role models in a video clip. Older adults in the intervention group reported a significant increase in self-reported weekly volunteering minutes six weeks after the intervention as compared to the active and passive control group [30].

The RCT by Warner et al. [30] was the first to show the feasibility of promoting volunteerism in older persons without prescribed task assignments. It gives, however, only limited insight into the working mechanisms of volunteering interventions and bears some limitations in measurement and intervention intensity, which should be addressed in the proposed RCT described in this study protocol: First, only short-term effects of the intervention were evaluated in the German RCT [30]. Second, mechanisms underlying the effect of the intervention on initiation and maintenance of volunteering were not investigated in the German RCT and remain largely unknown [30]. Third, the intervention in the German RCT [30] was of low intensity (one session) that may limit the possibility to find long-lasting effects. These shortcomings are addressed by the RCT described in this study protocol.

### Study objectives and hypotheses

The main hypothesis of the current RCT is that the volunteering intervention is effective to increase the primary outcome – self-reported minutes of volunteering per months – in Hong Kong and shows short-term (6 weeks after the intervention) as well as medium to long-term effects at three months and six months follow-up compared to an active control group, targeting physical activity.

To unravel the working mechanisms of the intervention based on the HAPA [29] and suggested by Warner et al. [30], the following active ingredients of the intervention are tested: outcome expectancies of engaging in volunteer work (perceptions of benefits through volunteer work), self-efficacy (the belief in being able to volunteer on a regular basis), intentions (different motives as well as concrete goals for volunteering), planning (e.g. the formation of implementation intentions), and self-monitoring (keeping track of volunteer work). As all of these constructs are explicitly targeted in the volunteering intervention group, we hypothesize that the intervention group will show increases in these cognitions and self-regulatory strategies as compared to the control group.

Secondary objectives of the study are to detect possible mechanisms of the intervention effect with further mediation analyses (sense of community, generative concern, perceived costs of volunteering as well as motives to volunteer) and to test the effect of increased volunteering on volunteers’ reports of depressive symptoms, subjective health, meaning in life, general self-efficacy, and perceived autonomy. To test for differential effects of possible subgroups within the intervention group, socio-demographic information, satisfaction with volunteer work, volunteering enjoyment, symptoms of volunteer burnout, religiousness, and attitudes towards older adults are assessed as moderators of intervention success (see measures section for details).

## Methods/design

### Participants and procedure

#### Research integrity

Ethical approval for this RCT was obtained by the Human Research Ethics Committee (HREC) of the Education University of Hong Kong (HREC number 2015–2016-0324). The study had been prospectively registered in the Clinical Trials Registry of the Center for Clinical Research and Biostatistics (CCRB) recommended by the World Health Organization (Primary Registry and Trial Identifying Number ChiCTR-IIC-17010349, secondary CCRB trial number CUHK_CCRB00543, www2.ccrb.cuhk.edu.hk/registry/public/416). The RCT is funded by the Public Policy Research (PPR) Funding Scheme, Policy Innovation and Co-ordination Office (PICO) of the Hong Kong Special Administrative Region Government (project number 2016.A5.023.16C).

This is the first version of the study protocol. No further study protocols will be published.

#### Recruitment and eligibility

A sample of community-dwelling adults aged 50 and older is recruited via invitation of local community centers within all districts of Hong Kong. The eligibility criteria are: 1) 50 years of age and older; 2) not acutely physically impaired or disabled; 3) not seriously cognitively impaired or severely depressed; 4) not working on a full-time or part-time basis (fewer than 20 h per week); 5) able to read and write simple Chinese; and 6) engaged in formal volunteer activities fewer than four times in the past year. Except for meeting the eligibility criteria there were no further exclusion criteria.

Through local community centers, participants are scheduled for one of several group orientation meetings held at their nearest center, during which details of the study’s purpose, time commitment involved in study participation, and inclusion criteria are explained. Individuals who meet the criteria are asked to sign a written informed consent form. The research team ensures confidentiality of this data by keeping questionnaire data and consent forms in different locked filing cabinets, to which only research assistants, who signed a data protection form have access. All participants are reimbursed with HKD 200 (approx. 25.50 USD or 22 EUR) in the form of a voucher for a supermarket if they complete all intervention sessions and all four assessment points. Participants cannot be blinded as it becomes obvious whether they are randomized to the experimental group targeting volunteering or the active control group targeting physical activity during the social-cognitive intervention.

#### Power analyses and sample size

The study by Warner et al. [30] found an intervention effect with an effect size of 0.38 (Cohen’s d). For the power calculation of the intervention effect in the proposed study, an estimated 15% dropout rate over the study period, a power of 90% and an alpha level of 5% is set. To detect this effect size using T-tests, a minimum sample size of 240 is needed.

The study is conducted in 15 centers belonging to two NGOs’ elderly centers, located in 9 out of 18 districts in Hong Kong. In each center, participants are assigned individually and at random to either the experimental or control group by the facilitator. Experimental and control groups are expected to run with 10 to 15 participants. Due to randomization within clusters, the statistical power might be slightly diminished; therefore, the aim is to recruit 360 instead of 240 participants in total.

#### Study design

At baseline, a research assistant or a part-time interviewer interviews participants in a face-to-face format using the structured questionnaire. The intervention period for both groups comprises 4 weeks, with one session lasting approximately 1 hr conducted each week at the respective senior centers. After the intervention and active control intervention period, participants are interviewed three times in a face-to-face format, at 6 weeks, 3 months, and 6 months after the intervention period. Intensive training is provided to the part-time interviewers, to ensure reliability.

#### Intervention procedures and delivery

In accordance with the volunteering intervention developed by Warner et al. [30], which serves as a basis for this refined intervention, and in lack of a social-cognitive theory specifically for volunteering, the well-established social-cognitive framework of the health action process approach (HAPA; [29]) is chosen as theoretical backdrop for intervention development. This model postulates that behavior change is based upon developing an intention, which is built upon risk-perception (not assumed to be relevant for volunteering), outcome expectancies, and self-efficacy in the motivational phase, whereas intentions are translated into behavior via self-regulatory strategies such as planning and self-monitoring in the volitional phase [29]. As non-volunteers are the target group of the current study, motivational as well as volitional (self-regulatory) strategies are chosen to support participants in the initiation as well as maintenance phase of their new volunteer activity.

#### Manualised intervention delivery

One experienced social worker and one research assistant are hired to deliver the intervention to the experimental group and the active control group conditions for all sessions, respectively. They learn all session protocols by heart and read the standardized intervention manual before every session. The research team provides them with intensive training with the manual and role-play exercises. Moreover, the sessions are structured using standardized PowerPoint presentations to ensure the comparability of session contents. To enhance fidelity to the protocol and quality of intervention delivery, at least five sessions in the intervention condition and five sessions in the active control condition are observed and rated by the fourth and fifth author of this manuscript.

#### Experimental condition

The following behavior change techniques (BCTs) are used in four interactive group intervention sessions to prompt volunteering and categorized according to Michie’s BCT taxonomy [30]: information about the benefits of volunteering in old age (prompt for outcome expectancies), focus on past success (prompt for self-efficacy in biography worksheet), goal setting behavior and outcome (prompt for intention formation in worksheet), action planning and use of cues (prompt for use of planning strategy in worksheet), modeling behavior (prompt for self-efficacy in a 5-min video clip with older person as role model). Furthermore, information material on volunteer opportunities for older adults in their residential area is available for free.

Four one-hour sessions are conducted: 1) introduction to the study and the meaning of retirement; 2) quiz regarding volunteer engagement; 3) ideas for volunteer engagement; 4) self-regulation techniques to translate intentions into behavior. These topics are delivered to participants by means of weekly one-hour sessions with two breaks.

In the first session, the facilitator briefly introduces the research team, the goals of the study, the target population, schedule, follow-up questionnaires, and financial incentive. An ice-breaking game gives group members the chance to get to know each other. The facilitator discusses the personal meaning of retirement with participants by asking them about their ideas (e.g., positive or negative connotations, retirement expectations, elements of their ideal retirement, favorite activities during their retirement). The facilitator writes down participants’ responses on a whiteboard to acknowledge their ideas; she also provides more ideas, such as the development of hobbies, activities with friends, activities with family, doing work around the flat, travelling, and cultural events. Finally, the facilitator introduces the idea that one further important aspect of retirement could be doing something for others or taking an active part in volunteering by mentioning the social relevance of volunteer activities as well (retaining knowledge, caring for those in need, strengthening social integration and older people’s role in society). Some individual benefits of volunteering (effects on physical and psychological health) and societal advantages as well as economic aspects are presented to participants at the end of this first session.

In the second session, the facilitator first discusses the positive outcomes of engaging in volunteer activities with the group members. The facilitator asks if participants expect any benefits from being volunteers, whether they perceive those benefits as being relevant to themselves, and their underlying reasons for wanting to volunteer. Information on the positive consequences of volunteering in old age is delivered through a quiz on volunteer engagement. The quiz includes information on the prevalence of volunteering in the general population in Hong Kong and among Hong Kong’s older adults, future trends in volunteering in old age, financial rewards for volunteer activities, reasons for engaging in or barriers for not engaging in volunteer activities (too old, too busy, poor physical health, etc.), and ways to volunteer (e.g., through NGOs, religious organizations, educational organizations, community organizations). The facilitator discusses how to overcome barriers for volunteering in old age with the group members. Finally, group members are asked to reflect on whether they want to be a volunteer, the reasons for this decision, and to share their thoughts with other group members.

The third session starts with a review of the first two sessions. Then, the facilitator shows two five-minute video clips in which older volunteers are presented as positive role models. In the video, a woman and a man introduce themselves as being over 70 years old. The man has problems walking, while the woman did not receive formal education. Nevertheless, the role models actively volunteer in social areas by visiting older people, who live alone and by teaching older adults how to use computers in senior centers. The models express how they love their volunteer work, because it makes them feel needed and that giving pleasure to others renders them pleasure as well. In the video, the models speak of their experiences with volunteering. The models explain how they started their work as volunteers, how they planned to volunteer, and how they enjoyed volunteering immensely. After showing the video chips, the facilitator asks participants to discuss what they feel towards the role models and the most important benefits of being a volunteer from their perspectives. Then, participants are asked to think about the areas in which they would like to be active volunteers. The facilitator provides a decision aid by showing different age groups as recipients of volunteer support, such as children, adolescents, adults, and older adults as well as some specific vulnerable groups, such as migrants, families with financial difficulties, people with disabilities, and people with chronic medical conditions. The facilitator introduces some concrete examples of volunteer work, such as escorting others, doing household chores, acting as receptionist, measuring blood pressure, fundraising, visiting other people, designing posters, taking photos, and reading newspapers or storybooks to others. Different categories of volunteering, including clerical work, elderly care, child care, and organizing activities, are also presented along with different venues for volunteer activities, such as community centers, social service organizations, hospitals, religious groups, cultural organizations, animal shelters, and political parties. Volunteering opportunities at the elderly center are briefly mentioned by the staff of the senior center hosting the intervention group. A list of volunteering opportunities in each district of Hong Kong and the websites to search for volunteer opportunities are presented to the group. Finally, the facilitator discusses the amount of time that participants would like to spend on volunteering and warns them that excessive volunteering may be detrimental to older adults’ health. Participants are informed that they should identify their optimal level of volunteering per week and should not exceed this level, to prevent burnout. Before the end of this session, participants set goals related to volunteer activities: 1) the organization; 2) the target group; 3) the nature of the volunteer activities; 4) personal goals; 5) activities or services; and 6) frequency of participation.

In the fourth session, participants are asked to focus on past experiences using a biography worksheet. On this worksheet, participants are invited to look back on different periods within their own biography. They are asked: 1) to state the hobbies and volunteer engagements they participated in during the course of their lifes; 2) whether they participated in these activities regularly; 3) whether they enjoyed performing these activities back then; and 4) for how long they engaged in these activities. The lifespan is divided into childhood and youth (under 20 years), young adulthood (20–30 years old), and adulthood (40–60 years old). After these recollections, participants are asked if they had engaged in volunteer activities based on their hobbies and about volunteer engagements in their late adulthood. The facilitator discusses different personal goals related to engaging in volunteer activities (e.g., helping others, doing something meaningful, spending time with others, self-actualization, use of skills or experiences, improving the community, religious reasons, and acquiring new skills or knowledge) and participants are asked to discuss their personal goals to engage in volunteer activities.

Once participants have established their personal goals and plans to volunteer, the facilitator introduces the Proper-Effective-Practicable-Plannable-Test (PEPP-Test, similar to setting SMART goals). In this test, participants are asked to think about a volunteer engagement that would suit them, whether they would enjoy this kind of engagement, and whether this engagement matches their areas of interest for the first P (Proper). In the E-test (Effective) participants are asked whether they think the chosen volunteer activity is effective to achieve their personal goals. In the third P-test (Practical) participants are asked, whether the plans they made are practicable and feasible. In the last P-test (Plannable) participants are asked whether their formulated volunteering plan fits within their weekly schedules and is not too demanding.

Afterwards, participants are asked to complete if-then implementation intentions on a worksheet, to initiate action planning and use of cues [31]. In this worksheet participants are asked to come up with different situations, objects, or people to which they could link a certain volunteering activity, using if-then sentences. Examples include “If I had breakfast, then I will go to the agency for volunteer engagement”; “If I see my calendar, then I will be reminded to look for a volunteer engagement”; “If I meet my friends, I will ask them about their experiences with volunteer engagements.” Participants are engaged in a discussion about tricks they could use on themselves to render becoming active volunteers easier. Besides the tricks participants mention themselves, the facilitator introduces a diary to help them self-monitor their progress towards more volunteering. In this diary, participants are asked to record whether they engage in volunteer activities and, if they do, the nature of the volunteer activities, the amount of time they dedicate to volunteer activities, and how they feel after carrying out or not carrying out planned volunteer activities (negative or positive) in the coming 6 months. In addition, a six-month calendar is provided, in which participants can schedule their volunteer appointments.

By the end of the last session, participants are asked to form a self-support group. Each group receives the opportunity to meet again at the respective senior center to share their volunteering experiences, the benefits of participating in volunteer activities, and any barriers or difficulties preventing them from participating in volunteer activities. The facilitator also asks them to name three significant others, who could support them in implementing their plans related to volunteering, to prompt social support.

#### Active control group

Staff members of the hosting senior center briefly introduce volunteering opportunities at their center to participants and provide them with the same list of volunteering opportunities in each district and information about websites to search for volunteer activities as in the intervention group. Apart from this brief and purely informational intervention content focusing on volunteering, the active control group receives the same behavior change techniques as the volunteering intervention group in four parallel interactive group sessions with similar length, but all material is adapted to increase physical activity. Physical activity is chosen as the target behavior for the active control group as it resembles volunteering in several aspects: 1) It has similarly positive consequences for mental as well as physical health and longevity among older adults if performed on a regular basis [32, 33]. 2) The recommended amount of physical activity is only reached by a minority of older adults in Hong Kong (only 29% of the adult population aged 65 and older engage in 150 min of physical activity per week as recommended by the World Health Organization; [34]). 3) And social-cognitive motivational and self-regulatory intervention techniques are well applicable to the initiation and maintenance of regular physical activity, ensuring that parallel intervention content can be set up for both group conditions [35]. The only difference in group session content is that no PEPP-test and no self-monitoring is performed in the physical activity group and that this group learns some mild stretching and strengthening exercises during the group sessions in addition to the social-cognitive intervention techniques to ensure that participants’ expectations of an exercise group are met and to keep attrition rates low.

The facilitator in the active control group is the same as in the intervention group. This facilitator is supported by an additional trainer for physical activity in the elderly, who leads the actual physical exercises in the active control group (stretching and low-impact cardio exercises). For a detailed description of the active control group as compared to the intervention group, see Additional file 1.

#### Measures

See Table 1 containing the SPIRIT information [36] for an overview of time-points and measures in this RCT.

**Table 1:** SPIRIT schedule of enrollment, interventions, and assessments [36]

	STUDY PERIOD								
	Enrolment	Allocation	Post-allocation						
TIMEPOINT	*-1*	Baseline (T0)	*Intervention period*				*6 weeks post-test (T1)*	*3 months follow-up (T2)*	*6 months follow-up (T3)*
			Week 1	Week 2	Week 3	Week 4			
ENROLLMENT:									
Eligibility screen	X								
Informed consent	X								
Allocation		X							
INTERVENTIONS:									
*Volunteering Intervention*			X	X	X	X			
*Active Control Intervention*			X	X	X	X			
ASSESSMENTS:									
*Socio-demographic variables*		X							
*Covariates*		X							
*Primary Outcome*		X					X	X	X
*Secondary Outcomes (including subjective health)*		X					X	X	X
*Mediator variables*		X					X	X	X
*Moderators variables*		X					X	X	X

#### Primary outcome

Self-reported minutes of formal volunteering minutes per month is chosen as the primary outcome measure as it has shown to have a reliable link to positive health outcomes in previous research (as compared to further possible outcomes such as the frequency of volunteering, or the number of organizations; [37]). The definition of volunteering – formal volunteering for organizations – is explained to participants before they answer the questionnaires. Monthly volunteering minutes are assessed at baseline and 6 weeks, 3 months, and 6 months after the completion of the intervention. Two items validated by Ayalon [38] and based on the study by Warner et al. [30] are used 1) “During the past four weeks, on how many days per week did you do volunteer work?” and 2) “If you did volunteer work, how many minutes did one session last on average?”. Sessions per week and minutes per session are multiplied to derive the average minutes of volunteering per month.

In addition to this primary outcome, further information on volunteering is assessed: 3) the type of volunteer activity “If you did volunteer work, what type of volunteer work did you perform?”, 10 categories (i.e., recreational, manual labor, keeping company, domestic, educational, caring in hospices, sociocultural, administrative, social, and managerial) based on the German Survey on Volunteering is provided as answering options [39]; 4) benefits of volunteering for younger generations are assessed using the item “Did the volunteer work you did in the past four weeks benefit younger generations?” with “yes” and “no” as answering options; and 5) “For how many organizations did you serve as a volunteer during the past four weeks?” is assessed with an open format.

#### Mediators

The hypothesized mediators and active ingredients of the social-cognitive intervention to increase volunteering derived from the HAPA model are perceived benefits of volunteering, self-efficacy, intentions, planning and self-monitoring.

*Perceived benefits of volunteering* are assessed with the Chinese version of the perceived benefits subscale developed by Warburton et al. [26]. The subscale consists of five items (e.g., feeling useful, helping those in need, being busy and active). These positive expectations are rated on seven-point bipolar scales ranging from 1 (extremely unlikely) to 7 (extremely likely).

*Self-efficacy for volunteering* is measured by Wang’s volunteering self-efficacy scale, which comprises three items [40]. An example item is “How much confidence do you have in your ability to participate in volunteer activities?”. All items are rated on a five-point scale from 1 (not at all) to 5 (an extreme amount).

*Intention* to volunteer is assessed with four items based on previous volunteer intention measures [40, 41]. An item example is “Do you agree that you have decided to participate in future volunteering?” Answers range from 1 (strongly disagree) to 5 (strongly agree).

In lack of specific scales to assess self-regulation for volunteering, *planning* and *self*-*monitoring* for volunteering are assessed by adapting scales from the physical activity domain developed by Sniehotta et al. [42]. An item example for one of the four action planning items is “I have made a detailed plan regarding when to do volunteer work”. The two items for self-monitoring are “During the last four weeks, I have constantly monitored myself whether I volunteer frequently according to my plans”; and “…watched carefully that I volunteer as often as I intend to”. Planning and self-monitoring items are rated on a six-point scale from 1 (not at all true) to 6 (exactly true).

To test for further motives and values that might change through the intervention, additional exploratory mediators are assessed at all measurement points in time.

The Chinese version of the Loyola Generative Concern Scale [43, 44] is used to measure *generative concerns* with 20 items, in which participants are asked to what degree items describe themselves on a four-point scale ranging from 0 (never) to 3 (very often). Example items are “I try to pass along the knowledge I have gained through my experience” and “I feel as though I have made a difference to many people”.

*Sense of community* is assessed using the 12-item Sense of Community Index [45], which has been translated into Chinese and validated in the local community [46]. Participants are asked to rate items on a four-point scale from 1 (strongly agree) to 4 (strongly disagree). Example items are “I think the street/housing estate that I am living in is a good place for me to live” and “Very few of my neighbors know me” (recoded).

The Volunteer Functions Inventory (VFI; [47]) is used in its Chinese version [48] to measure participants’ *motives to volunteer* (i.e., the expected outcomes of volunteering). Participants are asked to indicate how important or accurate each reason for volunteering is for them on a seven-point scale from 1 (not important or not accurate at all) to 7 (very important or very accurate). This scale comprises 30 items, measuring six volunteering motives with five items each: 1) values (providing opportunities to express values regarding humanitarian concerns for other vulnerable people and helping others); 2) understanding (providing opportunities to acquire knowledge, skills, and capacities through novel learning experiences); 3) career (providing opportunities to gain career-related benefits); 4) protection (providing opportunities to reduce feelings of guilt and to solve personal problems); 5) enhancement (providing opportunities to enhance one’s ego); and 6) social (providing opportunities to maintain or improve friendships and to engage in activities admired by peers or significant others).

Further *motives for volunteering* are assessed with the Pleasure and Pressure based Prosocial Motivation Scale [49]. This scale contains a 4-item Pleasure based Prosocial Motivation subscale (e.g., “Supporting other people makes me very happy.”) and a 4-item Pressure based Prosocial Motivation subscale (e.g., “I feel indebted to stand up for other people.”). Participants respond using a 5-point scale ranging from 1 (strongly disagree) to 5 (strongly agree).

*The perceived costs* of volunteering are assessed with 5 items from the perceived costs and benefits scale validated by Warburton et al. [26]. Perceived negative consequences of volunteering (e.g., being overcommitted, being taken for granted, being tied down) are rated on a 7-point bipolar scale ranging from 1 (extremely unlikely) to 7 (extremely likely).

#### Secondary outcomes

*Depressive symptoms* are assessed with the Geriatric Depression Scale-Short Form [50, 51]. Participants are asked to tick 1 (no) or 0 (yes) for whether they have experienced any of 15 symptoms within the past 2 weeks (e.g., “Have you dropped many of your activities or interests?”, “Do you feel that your life is empty?”).

*Meaning in Life* is assessed with the Chinese version of Steger’s scale [52, 53]. Items read for example “I have discovered a satisfying life purpose” and “I understand my life’s meaning.” and are rated on a scale from 0 (absolutely not true) to 6 (absolutely true).

Levels of *general self-efficacy* are measured with the validated Chinese version of the General Self-Efficacy Scale [54]. Item examples are “I can always manage to solve difficult problems if I try hard enough” and “If someone opposes me, I can find the means and ways to get what I want.” Items are rated from 1 (not at all true) to 4 (exactly true).

*Perceived Autonomy* [55] is assessed with a 4-item scale (e.g., “I live by my own choices now that I am old”, “I make my own decisions and don’t need others to protect me.”) and answered on a 4-point scale from 1 (strongly disagree) to 4 (strongly agree).

Some *physical activity related questions* are integrated into the questionnaire to test whether taking up volunteering activities also effects physical activities (diminishes vigorous sports activities and increases light leisure activities for example) or physical activity related cognitions. The Baecke Physical Activity Questionnaire [56, 57] is used to assess self-reported physical activity including light leisure time activities (“Do you or did you practice sports or physical exercise within the past 12 months?” and “Have you changed your habits on leisure activities within the past 12 months? (e.g., playing mahjong, morning exercises, watching TV, playing chess, reading, etc.)” followed by open fields to indicate the type of activity and options to tick how many hours per week. Barriers, pros and cons for exercising are assessed with the The Chinese Barriers to Exercise Scale (CBE scale) and the Chinese Outcome Expectations for Exercise (COEE) scale [57, 58]. Example items for the CBE scale are “The venue for exercising is too far” and “I feel embarrassed doing exercise in public” and for the COEE scale examples “Doing exercise makes you feel tired easily” and “Doing exercise makes you sick easier”. With answering options from 1 (disagree) to 5 (agree). *Exercise Self-efficacy* is assessed with items adopted from Marcus et al. [57, 59], such as “On a scale from 0-10, how much would you conform, that you will develop a habit of exercising for one year (at least three times a week for a minimum of 20 minutes at a time)?” and “Are you confident that you can do the following actions?” (0–100% to indicate confidence in walking, running slowly, carrying heavy items, climbing up stairs, doing sit-ups).

*Stages of change for physical activity* are assessed with one item and 5 answering options based on the stages of change of the Transtheoretical Model by Prochaska and DiClemente [57, 60] “In a typical week, do you do regular physical exercise – meaning at least three times 20 minutes of physical exercise per week?”. Answers can be given on a visual analog scale from 10 to 0 with verbal anchors at 10 ("I do regular exercise and have maintained it for more than 6 months"), 8 ("I do regular exercise but only started for no more than 6 months"), 5 ("I exercise sometimes"), 2 ("I don’t exercise regularly, but I’m thinking about starting to exercise within 6 months") and 0 ("I do not exercise regularly, nor do I consider starting to exercise within the next 6 months").

#### Possible moderators of the intervention effect

To determine, whether the volunteering intervention works better for individuals with a specific mindset or socio-economic background, several moderator variables are assessed. Participants, who report to have volunteered within the past 4 weeks, are asked to rate further cognitions and emotions towards their volunteering activity: *Satisfaction with volunteer work* is assessed with one item [61] “Overall, how satisfied are you with volunteer work” rated from 1 (extremely dissatisfied) to 10 (extremely satisfied). The Chinese version of the *Volunteer Satisfaction Index* [62] consisting of 26 items (e.g., “I receive help when I need while volunteering.”, “The actual conditions of volunteer work are consistent with my expectations.”) and answered on a scale from 1 (very dissatisfying) to 7 (very satisfying). *General volunteering enjoyment* is assessed with one item by Okun et al. [63] “Overall, how much do you enjoy volunteer work?” rated from 1 (not at all) to 5 (very enjoyable). More differentiated *emotional responses to volunteering* are assessed with a rating scale from 1 (not at all) to 7 (extremely) for five positive (rewarding, exciting, interesting, enjoyable, fulfilling) and four negative emotions (emotionally draining, frustrating, disappointing, depressing [64]). To assess possible *symptoms of volunteer burnout*, the Chinese version of the Maslach Burnout Inventory Human Service Survey Scale is administered [41]. Volunteers are asked to rate 22 items on a 5-point scale from 1 (disagree completely) to 5 (agree completely), e.g. “I feel emotionally drained from my volunteer work.” and “I worry that my volunteer work makes me indifferent.”

All participants answer two questions on their level of *religiousness*. The two items are “I consider myself to be a spiritual person” [65], “I live according to religious principles” [66] and are answered from 1 (strongly disagree) to 4 (strongly agree).

All participants *rate their attitudes towards older adults* with the Chinese version of the Aging Semantic Differential [67, 68], that presents 10 bipolar semantic adjectives together with the question “Please indicate how you perceive older adults” (e.g., independent versus dependent, busy versus idle, secure versus insecure) to be rated on a 7-point scale between the two opposing poles.

#### Covariates

All analyses are statistically controlled for the following covariates: age, sex, partner status (without versus with partner), education (high school degree versus no high school degree), subjective health (“In general, how would you rate your health today?” from 1 (very good) to 5 (very bad)), and number of chronic conditions (“Has a doctor ever diagnosed you with any of the following diseases?” followed by a list of 10 diseases, e.g. arthritis, heart disease, hypertension, and an open field for further chronic diseases) as these socio-demographic and health measures have repeatedly been shown to be associated with the amount of volunteering [9, 10]. Subjective health is assessed at each time point; the other covariates are assessed at baseline only.

#### Data monitoring

The data monitoring committee consists of the coauthors of this study protocol, who are all independent from the sponsor of the study and have no competing interests. The trial will be stopped once the planned *N* was reached, no adverse events or unintended effects are expected.

#### Preliminary statistical analyses

Descriptive statistics, correlations of all variables, attrition analyses (T-tests comparing socio-demographic information as well as the level of expected outcome measures and mediators in participants, who drop out, to participants, who stay in the study until the last point of measurement) and randomization tests (T-tests comparing socio-demographic information as well as the level of expected outcomes and mediators in participants randomized to the intervention group to participants randomized to the active control group) are performed in SPSS 24.

#### Statistical analyses of intervention effects

The intervention effect on the primary outcome measure is first tested with repeated measures analyses of variance (ANOVA) to test for a time * group interaction over the six weeks, three months and six months follow-up. Based on the repeated measures ANOVAs, effect size calculations are performed using Eta^2^.

For more refined analyses, latent change scores, modeling change in volunteering minutes from baseline to six weeks, three months, and six months is performed in Mplus 8 [69]. This latent change approach has several advantages over traditional variance analyses: It allows testing differences from baseline to all post-tests in a single model instead of multiple tests, has more power to detect treatment effects, is robust to non-normality, and provides information on individual variability and fit statistics [70, 71]. All models are statistically controlled for age, sex, marital status, education, subjective health, and number of chronic conditions. A conditional model with baseline as reference point is set up in accordance with Mun et al. [71]. The change scores are then regressed on group condition and on the above-mentioned control variables. If any of the control variables show significant interactions with the group condition on volunteering, subgroup analyses are performed to find possible moderators that increase or decrease the intervention effect. In the change models, effect sizes are calculated via *R*^2^. Missing values are imputed via full information maximum likelihood estimation (FIML) in Mplus, as FIML makes use of all available data in model estimation [72]. To account for the cluster-randomization referring to participants clustered in groups and centers, analyses are conducted in a multilevel framework.

#### Statistical analyses of the mediation effects

To test for possible mediators of the intervention effect, manifest path analyses with indirect effects are specified and tested with 95% bias-corrected confidence intervals resulting from 1000 bootstraps in Mplus 8. Respective mediators at six weeks and three months as well as total volunteering minutes per month at six months follow-up are regressed on their respective baseline scores, the above-mentioned control variables as well as on group condition to test for an indirect effect of group condition on volunteering via these mediators in separate models for each mediator.

#### Dissemination strategy

The research team will publish one primary outcome article, describing the effect of the social-cognitive intervention on volunteering minutes per month. This article will include process analyses to elaborate on the active ingredients of the intervention material. Mediators named in the measurement section of this protocol are tested in the resulting publication. Another publication will focus on the secondary gains achieved by the intervention, testing effects on all secondary outcomes named in the measurement section of this protocol. Exploratory analyses are conducted to test for possible moderators of the intervention effect. If any of the covariates or moderators named in the measurement section of this protocol make a meaningful contribution to explaining the effects of the intervention on the primary or secondary outcomes, these analyses may result in further publications to inform future interventions on subgroups that are more or less receptive to the intervention.

## Discussion

Volunteering does not only contribute to society by supporting social services and charitable organizations but it has also been linked to mental and physical health benefits for those individuals, who volunteer – especially in old age [9, 10, 73]. Since volunteering rates are comparatively low in Hong Kong, but the proportion of older adults steadily raising [2, 3], the present RCT aims at increasing volunteering among community-dwelling older adults by means of social-cognitive behavior change techniques implemented in four face-to-face group intervention sessions for volunteering against a parallel active control group. The intervention is based on a previously successful intervention developed to increase volunteering in retirees in Germany [30]. The German intervention was conceptualized as a one-shot group intervention session of approx. three hours and resulted in increases in volunteering at six weeks follow-up but not at two weeks after the intervention as compared to an active control group. The authors conclude that the self-regulation strategies taught in the intervention, such as forming implementation intentions to search for volunteer services that match one’s interests and self-monitoring of progress toward more volunteering need time to unfold [30]. Therefore, this intervention should be replicated and improved by conducting four one-hour group intervention sessions during a period of four consecutive weeks among community-dwelling retired older adults in Hong Kong in the current RCT.

The effect size of the German intervention was rather small. Given the relatively low dosage of the intervention, with only one session of on average less than 170 min, it is expected that the proposed RCT results in a stronger effect size than the former intervention. Time-lagged effects found by Warner et al. [30] should not appear in the proposed RCT in Hong Kong, as the intervention was stretched to comprise four weeks, which should give participants more time in between the sessions to find and organize a suitable volunteer activity.

It is hypothesized that the RCT described in this protocol is effective in producing longer lasting intervention effects, since the intervention group will have more time and more opportunities to exchange volunteering plans, experiences and social support in the process of finding and initiating a suitable volunteer position [74].

### Potential for implementation of results

Hong Kong does already observe a major challenge for health care services and welfare due to its rapid demographic change [2]. As the proportion of older adults in the general population is expected to continuously rise, it is of utmost importance to capture this source of social capital, which would help those in need in Hong Kong’s society and at the same time provide benefits for volunteers’ mental and physical health.

If the social-cognitive intervention designed to increase engagement in formal volunteer work is effective in Hong Kong, it could be introduced to the general older population. Participants in this study are recruited from community centers for older adults. By recruiting community centers as hosts for the intervention sessions, we place the intervention into a setting, where a subsequent implementation is feasible. Dissemination of the study’s findings as well as future interventions to increase volunteering can make use of the senior centers’ members and infrastructure. Through various collaborations with age-care (social and health care) practitioners, the research team is optimistic that training manuals and intervention protocols describing the intervention program can be disseminated into practice to further promote the implementation of this intervention to improve volunteering rates among older Hong Kong Chinese. The research team believes that the protocol would also provide important references to other Asian societies that are characterized by low volunteer participation rates such as Singapore [6]. As most industrialized nations face the challenge of demographic change, an effective volunteering intervention could help prevent some of the societal challenges (e.g., labor shortage, burden on health care and long-term care services, intergenerational solidarity) inherent in a rise of the proportion of older adults – not only in Hong Kong – but also in other societies facing population ageing.

## Additional file

Additional file 1: Intervention content for experimental and control group by session. Point by point description of the intervention content delivered to the experimental volunteering group and the active control group for physical activity by intervention session. (DOCX 18 kb)

## Abbreviations

ANOVA: Analyses of variance
BCTs: Behavior change techniques
CBE: The Chinese Barriers to Exercise Scale
CCRB: Clinical Trials Registry of the Center for Clinical Research and Biostatistics
COEE: Chinese Outcome Expectations for Exercise
EC: Experience Corps
EUR: Euro
FIML: Full information maximum likelihood estimation
HAPA: Health Action Process Approach
HKD: Hong Kong Dollars
HREC: Human Research Ethics Committee of the Hong Kong Institute of Education
NGO: Non-governmental organization
PEPP-Test: Proper-Effective-Practicable-Plannable-Test
PICO: Policy Innovation and Co-ordination Office of the Hong Kong Special Administrative Region Government
PPR: Public Policy Research Funding Scheme
RCT: Randomized Controlled Trial
SPIRIT: Standard Protocol Items: Recommendations for Interventional Trials
USD: US Dollars
VFI: Volunteer Functions Inventory
