# Supplementary material for: Study protocol of a multi-center RCT testing a social-cognitive intervention to promote volunteering in older adults against an active control
Source: BMC Geriatr. 2019 Jan 24;19:22. doi: 10.1186/s12877-019-1034-1 (PMC6345019; doi:10.1186/s12877-019-1034-1)
Supplement: Supplementary file 1 — Intervention content for experimental and control group by session. Point by point description of the intervention content delivered to the experimental volunteering group and the active control group for physical activity by intervention session. (DOCX 18 kb) [file 12877_2019_1034_MOESM1_ESM.docx]

Additional file 1

Intervention content for experimental and control group by session

Belong to “Study protocol of a multi-center RCT testing a social-cognitive intervention to promote volunteering in older adults against an active control”

| **Intervention session 1** |  |
| --- | --- |
| **Experimental group** | **Active control group** |
| 1. The facilitator introduces the research team, the goals of the study, the schedule of sessions, follow-up questionnaires and financial incentive to participants. 2. Ice breaking activity: Participants introduce themselves to each other. 3. The facilitator introduces the meaning of life during retirement and discusses this topic with participants. 4. The facilitator promotes the financial benefits of volunteer activities, and encourages participants to join volunteer work in the future. 5. The facilitator briefly concludes the content of the first session. | 1. The facilitator introduces the research team, the goals of the study, the schedule of sessions, follow-up questionnaire and financial incentive to participants. 2. Ice breaking activity: Participants introduce themselves to each other. 3. The facilitator introduces the meaning life during retirement and discusses this topic with participants. 4. The facilitator explains the disadvantages due to lack of physical activity, and encourages participants to do more physical exercise in the future. 5. The exercise tutor teaches participants stretching exercises focused on upper limbs. 6. The facilitator briefly concludes the content of the first session. |

| **Intervention session 2** |  |
| --- | --- |
| **Experimental group** | **Active control group** |
| 1. The facilitator introduces the benefits of joining volunteer activities, such as the expansion of the social network and increases in psychological health. 2. The facilitator asks participants to join the game: 5-question-quiz about volunteer activities. 3. The facilitator discusses the statistics of volunteer activities mentioned in the quiz, in order to raise participants’ interest to join volunteer activities. 4. The facilitator reminds participants to reflect why they would like to join volunteer work. 5. The facilitator briefly concludes the content of the second session. | 1. The facilitator encourages participants to be physically active on a regular basis. 2. The facilitator asks participants to join the game: 5-question-quiz about physical activities. 3. The facilitator reminds participants what should be aware during physical activity, such as warm up exercises. 4. The facilitator introduces the benefits of physical activity, such as psychological well-being and physical health. 5. The exercise tutor teaches participants stretching exercises focused on the middle body. 6. The facilitator briefly concludes the content of the second session. |

| **Intervention session 3** |  |
| --- | --- |
| **Experimental group** | **Active control group** |
| 1. A review of the first two sessions is conducted by the facilitator. 2. The facilitator shows a five-minute video clip in which two older volunteers are presented as role models, in order to prove that older participants can join volunteer activities. 3. The facilitator introduces different volunteer target groups and different types of volunteer activities. In order to let participants choose what kind of volunteer activities they would like to try. 4. Participants are informed that they should identify their optimal level of volunteer work and not exceed this level to prevent burnout. 5. The facilitator asks participants to set their own goals related to volunteer activities before the end of the session. | 1. A review of the first two sessions is conducted by the facilitator. 2. The facilitator shows a five-minute video clip in which two older adults are presented as role models for physical exercise, in order to prove that older participants can join physical activities. 3. The facilitator introduces different types of physical exercises suitable for older adults and different kinds of sports venues. 4. Participants are informed that they should identify their optimal level of physical exercise and they should not exceed this level to prevent negative consequences. 5. The exercise tutor teaches participants stretching exercises focused on the lower limbs. 6. The facilitator asks participants to set their own goals related to physical activities before the end of the session. |

| **Intervention session 4** |  |
| --- | --- |
| **Experimental group** | **Active control group** |
| 1. The facilitator asks participants to recall what kind of skills or hobbies they had during their lifespan. Participants are asked if they could engage in volunteer work based on these past experiences. 2. Participants are asked to discuss their goals on volunteer activities and whether their goals are proper, effective, practical and plannable. 3. Participants are asked to complete the if-then implementation intentions in the worksheet for volunteering. 4. Participants are asked to discuss the tricks they can use on themselves, to make becoming active volunteers easier and the facilitator leads the discussion among participants. 5. The facilitator delivers a handbook to each participant and asks them to record their volunteering activities in it, in order to help them self-monitor their volunteering activities. 6. The facilitator briefly summarizes the content of all sessions and concludes the group. | 1. The facilitator asks participants to recall what kind of physical activities they engaged in during their lifespan. Participants are asked if they can re-engage these physical activities based on their past experience. 2. Participants are asked to discuss their physical activity goals and whether their goals are proper, effective, practical and plannable. 3. Participants are asked to complete the if-then implementation intentions in the worksheet for physical activity. 4. Participants are asked to discuss the tricks they can use on themselves, in order to help them do more physical activity, and the facilitator leads the discussion among participants. 5. The exercise tutor helps participants reviewing which exercises they have learnt in the previous sessions. 6. The facilitator briefly summarizes the content of all sessions and concludes the group. |
